# Parallel Battles: Managing Synchronous Cervical and Triple-Negative Breast Cancers

**DOI:** 10.7759/cureus.70201

**Published:** 2024-09-25

**Authors:** Fadila Kouhen, Malak Chahid, Reyzane El Mjabber, Najwa Benslima, Youssef Mahdi

**Affiliations:** 1 Radiotherapy, International University Hospital Sheikh Khalifa, Mohammed VI University of Sciences and Health (UM6SS), Casablanca, MAR; 2 Radiology, International University Hospital Sheikh Khalifa, Mohammed VI University of Sciences and Health (UM6SS), Casablanca, MAR; 3 Pathology, National Institute of Oncology, Rabat, MAR

**Keywords:** breast cancer, cervical cancer, individualized treatment, multidisciplinary approach, multiple primary malignancies

## Abstract

Multiple primary malignancies (MPMs) present diagnostic and therapeutic challenges, especially given their rarity and the distinct treatment strategies required. We report a case of a 66-year-old postmenopausal woman who presented with synchronous triple-negative breast cancer (TNBC) and cervical cancer, an uncommon and complex clinical scenario.

Given the complexity of her condition, a comprehensive multidisciplinary approach was employed. The treatment strategy included neoadjuvant chemotherapy for breast cancer, followed by breast-conserving surgery and axillary lymph node dissection. Simultaneously, cervical cancer was addressed with concurrent chemoradiotherapy and brachytherapy.

Remarkably, the patient demonstrated complete pathological responses in both the breast and cervical tumors following treatment. At 26 months of follow-up, she remains free of disease recurrence.

This case highlights the challenges of managing synchronous TNBC and cervical cancer. It underscores the necessity for individualized treatment plans and seamless multidisciplinary collaboration to achieve optimal patient outcomes.

## Introduction

Multiple primary malignancies (MPMs) occur when a patient develops two or more distinct primary cancers, each originating independently, rather than as metastasis or recurrence of the other. Although rare, MPMs pose considerable diagnostic and therapeutic challenges, requiring a nuanced and individualized approach to treatment [1].

An illustrative example is the synchronous occurrence of triple-negative breast cancer (TNBC) and cervical cancer [2]. TNBC is a particularly aggressive form of breast cancer that lacks the receptors commonly targeted in breast cancer therapies, making it more difficult to treat. Simultaneously, cervical cancer, originating in the cervical cells, brings its own set of clinical challenges and distinct treatment protocols. The simultaneous diagnosis of these two malignancies necessitates a carefully coordinated, multidisciplinary approach to develop a treatment plan that effectively targets both cancers without compromising the full therapeutic efficacy against each.

Risk factors for triple-negative breast cancer (TNBC) include genetic mutations, particularly in the BRCA1 and BRCA2 genes, as well as lifestyle factors like alcohol consumption and obesity. Reproductive history, such as early menstruation and late menopause, also plays a role [3]. TNBC is more prevalent among younger women and certain ethnic groups, particularly African American women. On the other hand, cervical cancer is primarily linked to persistent infection with high-risk human papillomavirus (HPV) types, especially HPV 16 and 18. Its risk is further influenced by factors like sexual behavior, immunosuppression, and lack of regular screening [4]. These distinct etiological pathways make the simultaneous occurrence of TNBC and cervical cancer rare, posing a unique clinical challenge. When these two malignancies do present together, they demand a comprehensive, coordinated treatment approach to effectively address both cancers.

This case report discusses the clinical presentation, diagnostic challenges, and therapeutic approach employed in managing a patient with these concurrent malignancies.

## Case presentation

A 66-year-old postmenopausal woman presented with a five-month history of spontaneous, moderately heavy metrorrhagia. The bleeding was not associated with urinary or gastrointestinal symptoms. Her overall health remained stable, with an ECOG performance status of 1. Her medical history was notable for a successfully treated episode of extra-pulmonary tuberculosis approximately 26 years earlier, with no recurrence. She denied experiencing pelvic pain, vaginal discharge, weight loss, fever, or any clinical signs of anemia.

Importantly, she had no family history of cancer and no identified risk factors for HPV infection, such as multiple sexual partners, immunosuppression, a history of other sexually transmitted infections (STIs), or smoking. Given the persistence of her symptoms, the patient consulted the gynecologist for further evaluation and management of her condition. During the physical examination, a 5 cm ulcer-bulging mass was identified on the cervix, extending to the upper third of the vagina as observed during the speculum examination. A subsequent bimanual examination confirmed that the parametrial invasion is distal. The rectovaginal examination revealed vaginal infiltration without invasion of the rectovaginal septum observed. No palpable lymphadenopathy was detected during the lymph node assessment.

**Figure 1:**
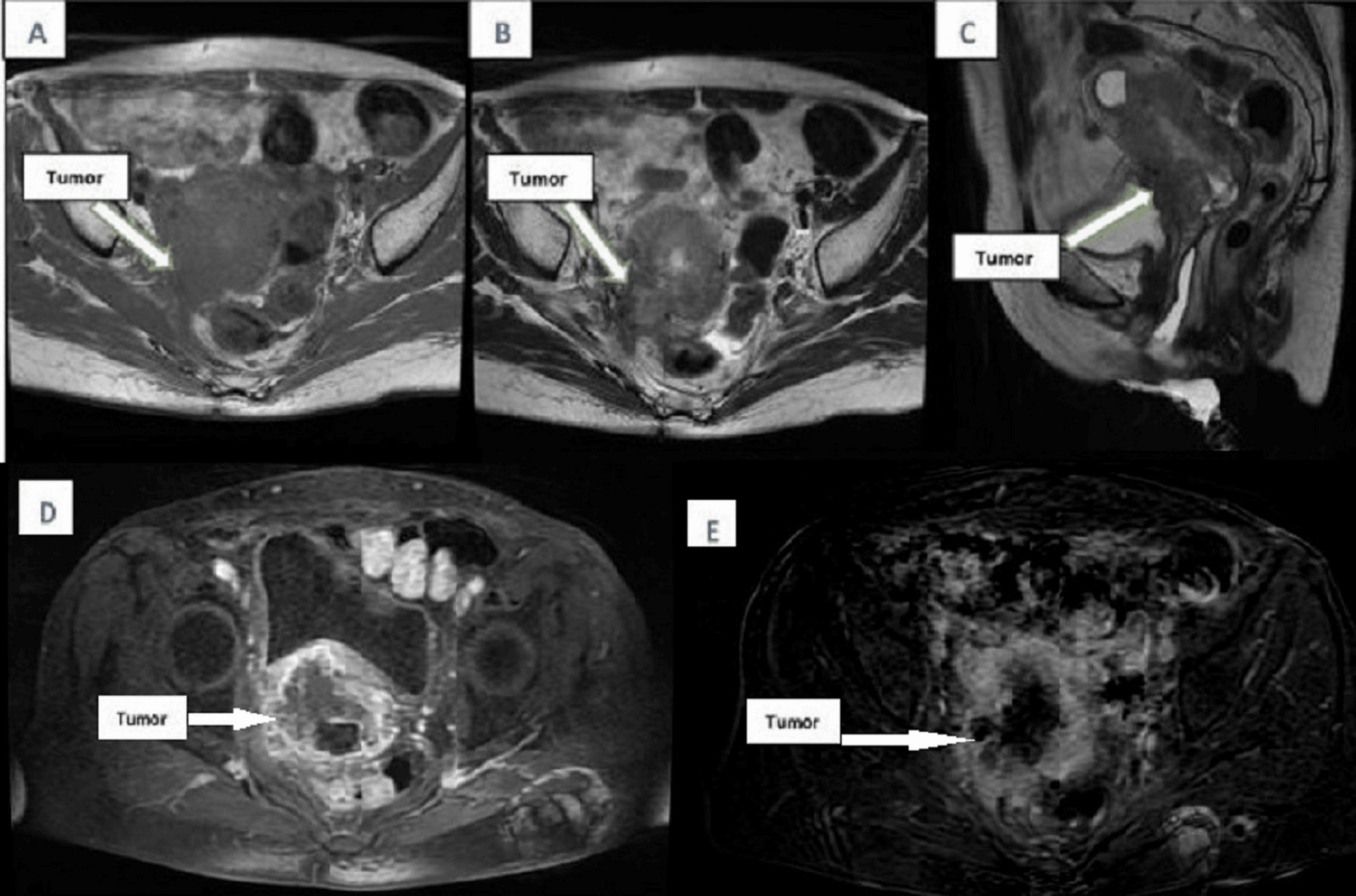
The tumor exhibited T1 hypointensity (A), T2 hyperintensity (B and C), with heterogeneous enhancement following gadolinium contrast administration (D), and heterogeneity on diffusion imaging (D). The mass extended into the upper third of the vagina and infiltrated the adjacent parametrial tissues.

MRI imaging revealed a significant mass in the cervico-isthmic region of the uterus, measuring approximately 58 x 55 x 34 mm. The tumor demonstrated T2 hyperintensity and T1 hypointensity, with heterogeneous enhancement following gadolinium contrast administration. This mass extended into the upper third of the vagina and infiltrated the adjacent parametrial tissues (Figure 1).

The lesion was in contact with the anterior rectal wall, without evidence of invasion into the rectal wall. It also abutted the posterior bladder wall, with the intervening fat plane remaining intact, indicating no invasion into the bladder. Bilateral external iliac lymphadenopathy was noted, with nodes measuring 9 mm on the right and 7 mm on the left, accompanied by adjacent fat infiltration. The Douglas pouch was free of any pathological involvement.

A cervical biopsy was performed, and histological examination revealed significant cellular atypia, characterized by large, irregularly shaped cells with hyperchromatic nuclei and a high nuclear-to-cytoplasmic ratio. High mitotic activity was observed, indicating rapid cell proliferation. The tumor cells exhibited an infiltrative growth pattern, invading surrounding tissues without forming the well-defined keratin pearls typically seen in more differentiated squamous cell carcinomas. These findings confirmed the diagnosis of a poorly differentiated squamous cell carcinoma.

**Figure 2:**
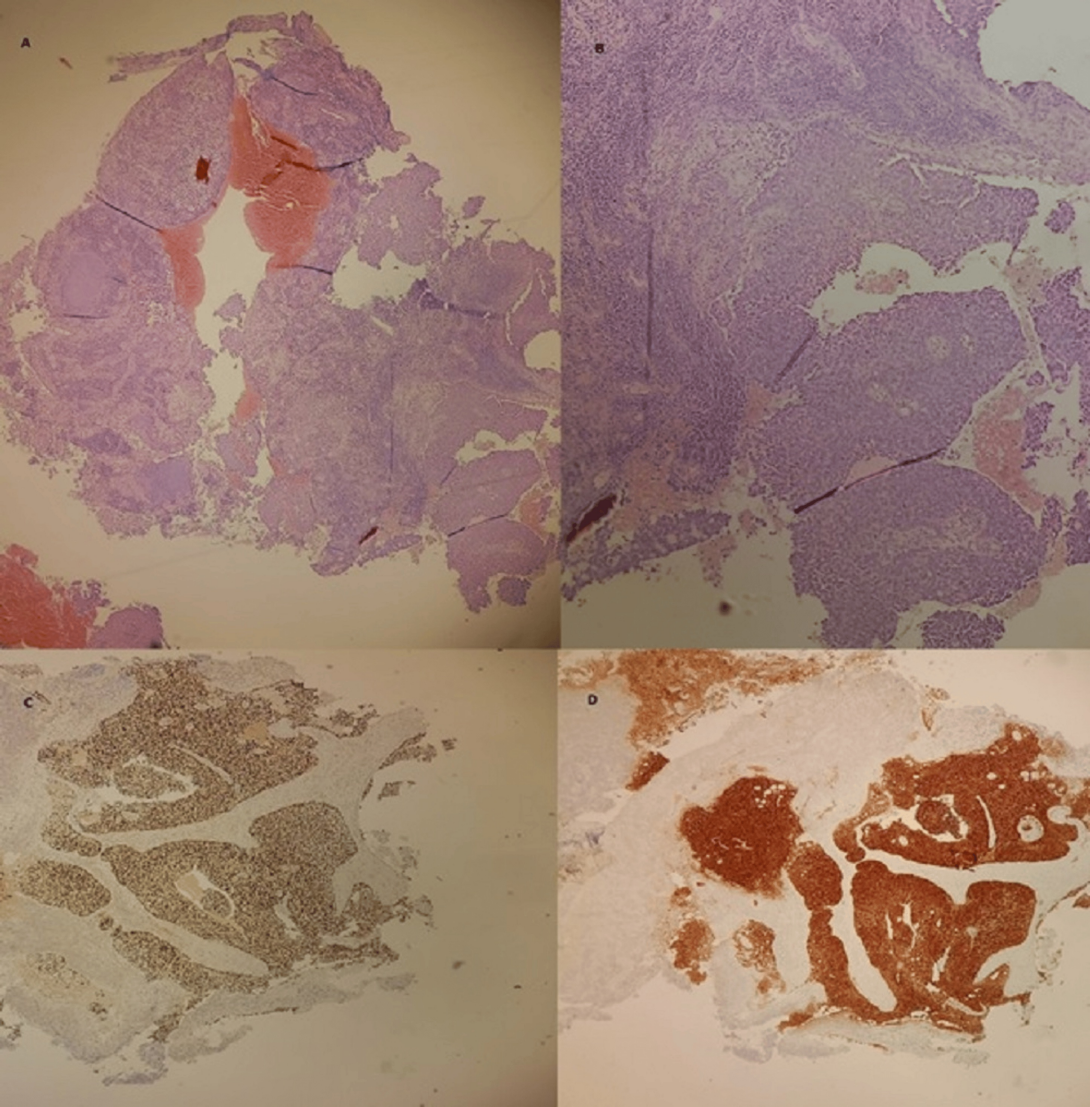
Representative micrograph of the cervical tumor. Lesion shows undifferentiated tumor proliferation (A). Tumor cells appear high grade with large nuclei and high nucleus to cytoplasm ratio (B). (Hematoxylin-eosin; A: x40, B: x100). The tumor cells are positive for p40 (C) and p16 (D).

**Figure 3:**
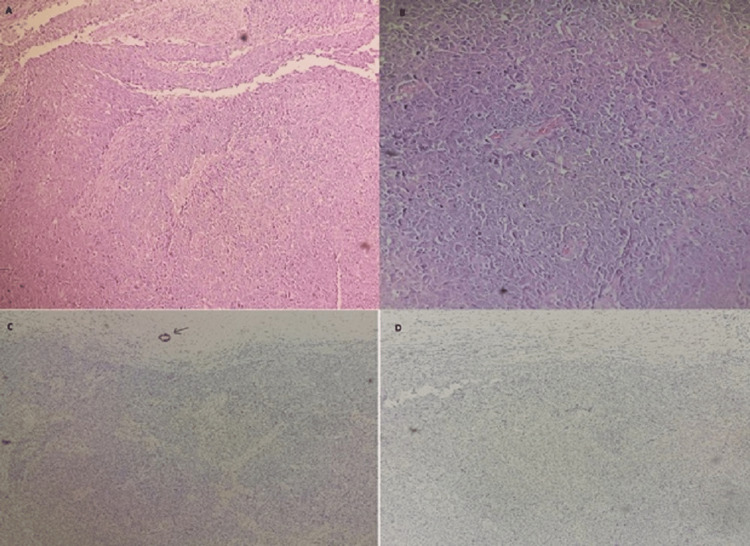
Representative micrograph of the breast tumor. Lesion shows high-grade invasive breast cancer of no special type (A). Tumor cells present large nuclei, marked variation and prominent nucleoli (B). (Hematoxylin-eosin; A: x100, B: x200). The tumor cells are negative for estrogen receptors (C) (arrow: positive staining of normal epithelial cells) and HER2 (D).

Immunohistochemistry further characterized the tumor, showing positivity for p16, CK5/6, and p63, which confirmed its squamous cell origin and suggested a potential association with HPV infection. A high Ki-67 proliferation index was noted, indicative of aggressive tumor growth. Importantly, the tumor was negative for estrogen receptor (ER) and progesterone receptor (PR) (Figures 2, 3).

**Figure 4:**
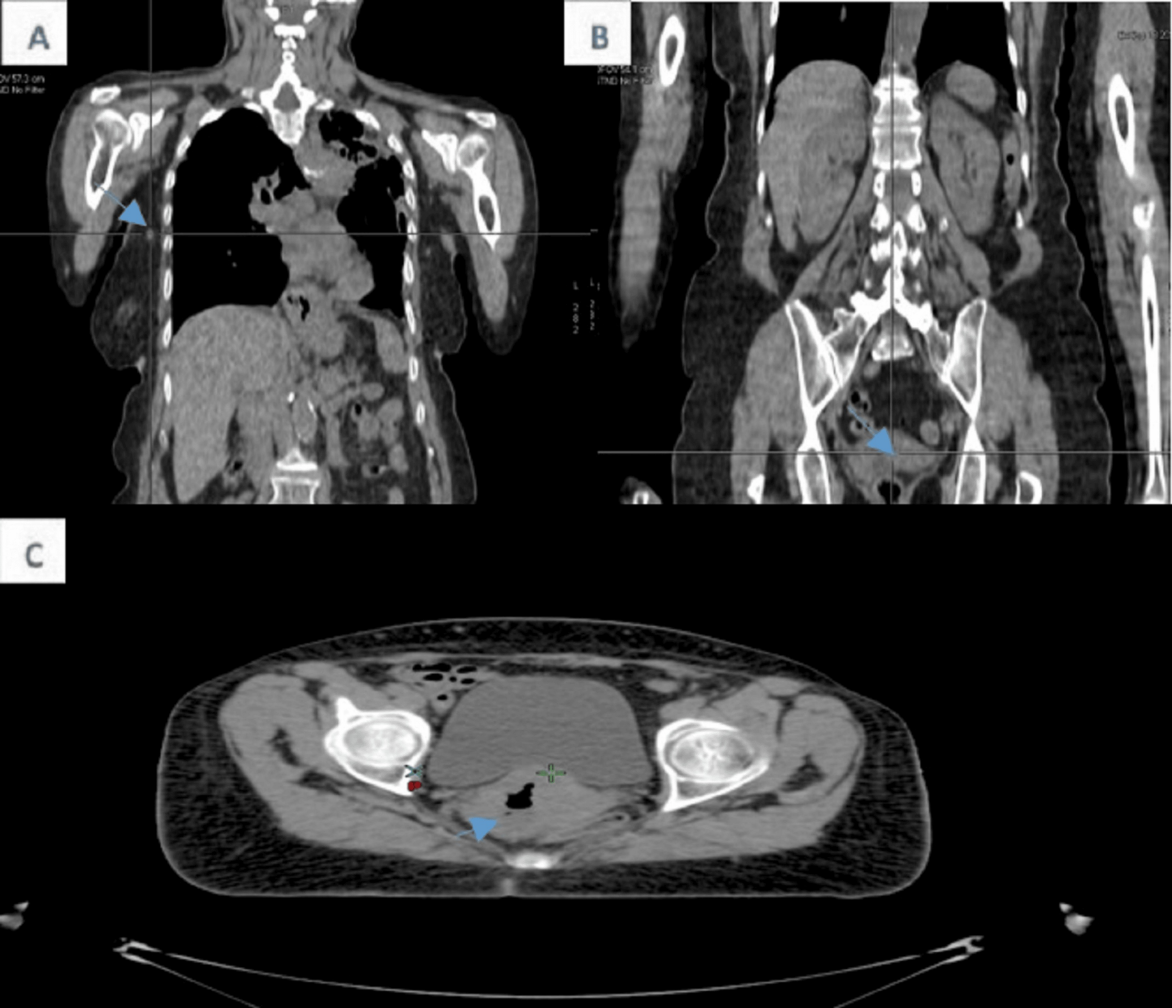
Staging CT scan: Coronal reconstruction reveals axillary lymphadenopathy (A) and cervical neoplastic process (B), with the axial slice illustrating the cervical tumor (C) that obliterates the fat plane where it abuts the anterior rectal wall. No evidence of distant metastasis was detected.

A staging CT scan was performed to assess the extent of the disease. The scan revealed four right axillary lymph nodes, with the largest measuring 13x17 mm. Additionally, a heterogeneous, hypodense mass was observed in the cervico-isthmic region of the uterus, consistent with previous imaging findings. The mass extended into the retro-uterine space, encroaching on surrounding structures. Notably, the tumor obliterated the fat plane in certain areas where it abutted the anterior rectal wall. However, no abnormalities were detected in the bladder or bones, suggesting no evidence of metastatic spread to these areas at the time of the scan (Figure 4).

**Figure 5:**
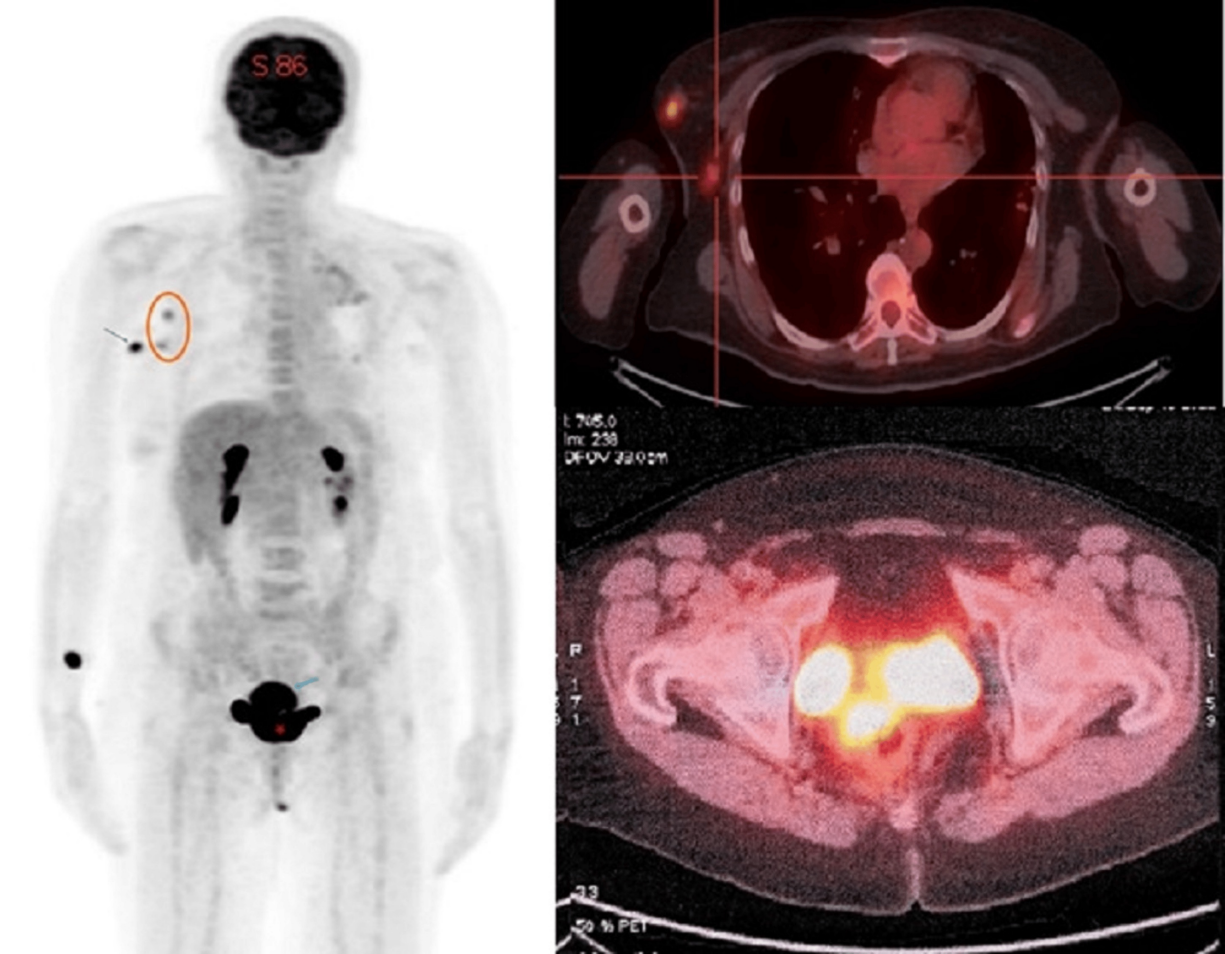
The 18F-FDG PET/CT shows simultaneous hyperfixation of the breast nodule with ipsilateral axillary lymphadenopathy and the cervical mass, without evidence of suspicious distant lesions.

Subsequently, an 18F-FDG PET-CT scan was conducted for comprehensive staging and to evaluate for any additional malignancies. The PET-CT scan revealed significant hypermetabolic activity in both the cervix and the right breast, each characterized by high standardized uptake values (SUVs), strongly suggesting the presence of malignancies in both locations. Furthermore, the PET-CT scan showed the involvement of the right axillary lymph nodes, which also exhibited increased metabolic activity, consistent with the findings from the CT scan. Importantly, no distant lesions were detected (Figure 5).

**Figure 6:**
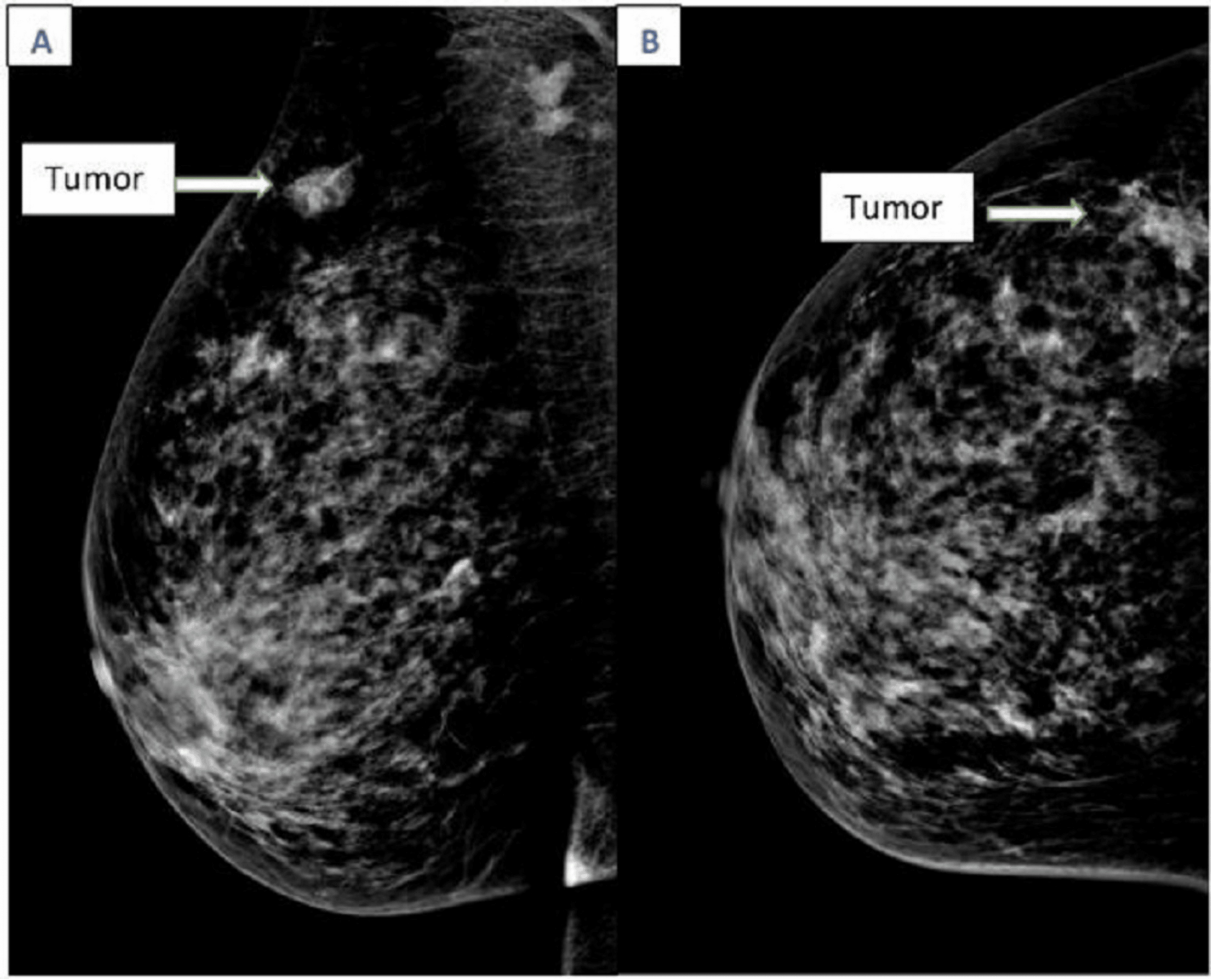
Mammography showed a nodular opacity with lobulated contours in the upper outer quadrant of the right breast, classified as ACR5 Ma (ACR 5) (A: craniocaudal view, B: mediolateral oblique view).

Mammography confirmed a 1.3 cm nodular opacity with lobulated contours in the upper outer quadrant of the right breast highly suspicious for malignancy and was classified as BIRADS 5 (Figure 6). The mammogram of the left breast showed benign findings, classified as BIRADS 2.

Following these results, an ultrasound-guided biopsy of the right breast was performed. Histopathological examination confirmed the presence of invasive ductal carcinoma, NOS (Not Otherwise Specified) type, graded as SBR grade III, indicative of a high-grade tumor. Immunohistochemical analysis revealed a triple-negative profile, with the tumor cells lacking expressions of estrogen receptor (ER), progesterone receptor (PR), and HER2 (human epidermal growth factor receptor 2). The Ki67 index, a marker of cell proliferation, was 15%, reflecting moderate proliferative activity. Additionally, the tumor cells were positive for Gata3, a marker supporting the diagnosis of a primary breast carcinoma origin. In summary, the patient’s final diagnosis was synchronous cervical carcinoma stage II B and right breast cancer that is HER2-negative, ER-negative, and PgR-negative T1b N1M 0.

The case was reviewed at a multidisciplinary team (MDT) meeting to devise a comprehensive evaluation and to develop a coordinated treatment plan. The treatment plan was designed to address both the cervical and breast cancers. It included radiation therapy with a dose of 46 Gy to the clinical target volume (CTV) for cervical cancer, complemented by a boost to specific areas. Additionally, high-dose rate (HDR) brachytherapy was administered to the residual cervical tumor, delivering a total dose of 28 Gy in four sessions of 7 Gy each. Due to the lack of reimbursement for pembrolizumab (Keytruda) in Morocco for both cervical cancer and triple-negative breast cancer (TNBC), a weekly chemotherapy regimen was implemented: Paclitaxel at 75 mg/m² and Cisplatin at 40 mg/m², both administered intravenously once a week for six cycles. Anthracyclines were excluded from the regimen. The paclitaxel and cisplatin regimen served as neoadjuvant therapy for breast cancer and concomitant therapy for cervical cancer. Before initiating chemotherapy, a radiopaque marker was placed at the site of the breast tumor under ultrasound guidance. Following completion of the chemoradiotherapy and brachytherapy, the patient underwent breast-conserving surgery with ipsilateral axillary lymph node dissection.

Histological examination revealed a complete pathological response with no residual tumor identified in the breast tissue. Additionally, thorough axillary lymph node dissection did not reveal any evidence of lymph node metastasis, classifying the Residual Cancer Burden (RCB) as 1.

The radiotherapy for the whole right breast, chest wall, and right supraclavicular fossa was delivered using a monocentric approach with a 3D field-in-field technique, administering a total dose of 42 Gy in 15 fractions of 2.8 Gy each. In light of the patient's age and the comprehensive treatment strategy, an additional boost to the tumor bed was deemed unnecessary.

Six weeks after completing radiotherapy for cervical cancer, a follow-up pelvic MRI confirmed a complete treatment response, with no residual tumor detected. Given the absence of clinically or radiologically detectable disease and the risk of fistula formation post-radiotherapy, no re-biopsy of the cervical tissue was performed, and further surgical intervention was not required.

During the follow-up period, the patient underwent systematic clinical and radiological evaluations to monitor treatment response and detect any signs of local or distant recurrence. This included annual pelvic MRI, contrast-enhanced CT scans of the thorax, abdomen, and pelvis (TAP), and mammography. After the 26-month follow-up, no evidence of disease recurrence was detected for both cancers, affirming the successful outcome of the treatment regimen.

## Discussion

Advancements in cancer screening, diagnosis, and treatment have significantly extended patient survival, leading to an increasing number of cases where individuals develop multiple primary neoplasms (MPNs) during their lifetime. The concept of multiple primary malignant neoplasms (MPMNs) dates back to the late 19th century, first introduced by the eminent surgeon Theodor Billroth [5]. Since, numerous cases of patients diagnosed with two or even three primary malignant neoplasms have been reported. Warren and Gates established specific criteria to identify multiple primary tumors accurately [6]. According to their guidelines: (1) Each tumor must be histopathologically confirmed to ensure that each is a separate primary malignancy. (2) The tumors must be geographically distinct and separated by normal tissue or mucosa, ensuring that they are not continuous extensions of the same tumor. (3) The possibility that one tumor is a metastasis of the other must be conclusively excluded. These criteria are essential for differentiating multiple primary tumors from metastatic or recurrent disease, which is critical for determining appropriate treatment strategies.

MPNs are defined as two or more distinct primary cancers that arise independently in the same patient, either at the same time (synchronously) or at different times (metachronously), without any one being a metastasis or recurrence of another [7]. According to the International Agency for Research on Cancer (IARC), synchronous neoplasms are those identified simultaneously or within a short interval of each other, typically within six months. In contrast, metachronous neoplasms are diagnosed more than six months apart [8]. Unlike secondary cancers that result from the spread of an initial tumor, MPNs originate separately, each with its own pathogenesis and clinical course. This distinction is crucial as it necessitates unique diagnostic evaluations and tailored therapeutic strategies for each primary malignancy.

Patients with a primary tumor are at a higher risk of developing additional primary cancers compared to the general population [9]. The development of multiple primary malignant neoplasms (MPMNs) is driven by several key factors [10]. Intrinsic factors include immune system weaknesses and hormonal imbalances, making the body more susceptible to new cancers. Extrinsic factors, such as prolonged exposure to environmental pollutants, ultraviolet radiation, and lifestyle choices like smoking and alcohol consumption, also contribute to the risk. Genetic factors play a role too, with inherited mutations like BRCA1 and BRCA2 increasing susceptibility to certain cancers. Additionally, therapeutic factors from prior cancer treatments, including radiation and chemotherapy, can induce new malignancies.

The risk of developing a second primary cancer after a breast cancer (BC) diagnosis is significant, with an incidence ranging from 4% to 16%. Among the most common secondary cancers are thyroid cancer and gynecological malignancies, particularly ovarian and endometrial cancers [11].

The current literature highlights the rare co-occurrence of breast cancer (TNBC) and cervical cancer, which is likely due to their distinct predisposing factors [12]. While cervical cancer is strongly linked to human papillomavirus (HPV) infection, a recognized primary risk factor, the association with breast cancer remains uncertain and lacks definitive confirmation. Some hypotheses suggest that HPV infection might potentially influence breast cancer development through mechanisms involving genetic alterations over time, possibly interacting with other environmental factors to promote carcinogenesis [13]. However, the association between HPV and breast cancer remains uncertain and lacks definitive confirmation. Conflicting research findings exist regarding the presence of HPV DNA in breast cancer samples, and the precise mechanisms of HPV transmission to breast tissue remain unclear.

The simultaneous presence of triple-negative breast cancer (TNBC) and cervical cancer presents significant treatment challenges due to their distinct biological characteristics and optimal treatment protocols [14].

TNBC is aggressive and lacks estrogen receptor (ER), progesterone receptor (PR), and HER2/neu receptor expression, limiting targeted therapy options that are effective for other breast cancer subtypes. Treatment typically involves a combination of surgery, chemotherapy, and radiation therapy. However, responses to chemotherapy can be unpredictable, and ongoing research aims to discover more effective treatment strategies. Encouragingly, the Keynote-522 trial [15] revealed a breakthrough: patients with early TNBC who received pembrolizumab combined with neoadjuvant chemotherapy experienced a significantly higher rate of pathological complete response compared to those treated with placebo plus neoadjuvant chemotherapy. This promising result underscores the potential of integrating pembrolizumab into treatment plans to enhance the chances of achieving a complete pathological response and improving outcomes for TNBC patients. In Morocco, pembrolizumab (Keytruda) is not reimbursed for either cervical cancer or triple-negative breast cancer (TNBC). This lack of reimbursement limits its accessibility for our patient, making it challenging to consider this treatment option despite its potential benefits in clinical trials.

On the other hand, cervical cancer treatment depends on factors such as the stage and type of cancer, but commonly involves surgery, radiation therapy, and chemotherapy [16]. The presence of HPV may influence treatment decisions, and recent advancements in immunotherapy have shown promise in certain cases.

The combination of breast and cervical cancer is exceptionally rare, with limited case reports in the literature and no established standard treatment protocols. Treatment approaches are often tailored based on local practices. Plachta et al. [17] in 2017 analyzed a case series of 200 patients with stage IIB-IIIB cervical cancer who underwent PET-CT for treatment planning. Among these, 2% had synchronous cancers (four cases), including two cases of breast cancer. For the first patient, the treatment regimen included neoadjuvant chemotherapy with paclitaxel and cisplatin (six cycles), followed by breast-conserving surgery with sentinel node biopsy. The cervical cancer was treated with radical radiotherapy and two hyperthermia procedures. Adjuvant therapy comprised breast cancer radiotherapy. The second patient received radical radiochemotherapy for cervical cancer with cisplatin and two hyperthermia procedures, followed by breast-conserving surgery with axillary lymph node dissection.

Managing both cancers simultaneously requires a multidisciplinary approach involving oncologists, surgeons, radiation oncologists, and other specialists. Coordinating treatment plans is crucial to ensure that therapies for each cancer do not compromise the effectiveness of treatments for the other. Additionally, managing side effects, monitoring for disease progression or recurrence, and providing supportive care are integral parts of comprehensive management.

## Conclusions

This case highlights the complexity of managing synchronous malignancies, especially involving aggressive histologies such as triple-negative breast cancer and advanced cervical cancer. The patient’s favorable response to neoadjuvant chemotherapy for breast cancer and concurrent chemoradiotherapy for cervical cancer emphasizes the critical role of personalized, multidisciplinary treatment approaches. Regular follow-up and monitoring are vital to ensure continued disease control and manage any long-term treatment effects.
